# Factors influencing traffic accident frequencies on urban roads: A spatial panel time-fixed effects error model

**DOI:** 10.1371/journal.pone.0214539

**Published:** 2019-04-04

**Authors:** Wencheng Wang, Zhenzhou Yuan, Yang Yang, Xiaobao Yang, Yanting Liu

**Affiliations:** 1 Key Laboratory of Transport Industry of Big Data Application Technologies for Comprehensive Transport, Beijing Jiaotong University, Beijing, China; 2 Department of Civil and Environmental Engineering, Pennsylvania State University, University Park, PA United States of America; 3 Department of Civil and Environmental Engineering, University of Washington, Seattle, WA, United States of America; 4 Key Laboratory of Road and Traffic Engineering of Ministry of Education, Tongji University, Shanghai, China; University of British Columbia, CANADA

## Abstract

China's rapid urbanization and high traffic accident frequency have received many researchers’ attention. It is important to reveal how urban infrastructures and other risk factors affects the traffic accident frequency. A growing amount of research has examined the local risk factors impact on traffic accident frequency at certain time. Some studies considered these spatial influences but overlooked the temporal correlation/heterogeneity of traffic accidents and related risk factors. This study explores risk factors’ influence on urban traffic accidents frequency while considering both the spatial and temporal correlation/heterogeneity of traffic accidents. The study area is split into 100 equally sized rectangle traffic analysis zones (TAZs), and the urban traffic accident frequency and attributes in each TAZ are extracted. The linear regression model, spatial lag model (SLM), spatial error model (SEM) and time-fixed effects error model (T-FEEM) are established and compared respectively. The proposed methodologies are illustrated using ten-month traffic accident data from the urban area of Guiyang City, China. The results reveal that the time-fixed effects error model, which considers both spatial and temporal correlation/heterogeneity of traffic accidents, is superior to other models. More traffic accidents will happen in those TAZs that have more hospitals or schools. Moreover, hospitals have a greater influence on traffic accidents than schools. Because of the location in the margin of the city, those TAZs that have passenger stations have more traffic accidents. This study provides policy makers with more detailed characterization about the impact of related risk factors on traffic accident frequencies, and it is suggested that not only the spatial correlation/heterogeneity but also the temporal correlation/heterogeneity should be taken into account in guiding traffic accident control of urban area.

## Introduction

In the field of transportation, the road traffic accident is a critical problem that urban road traffic must face. Over the past several decades, China’s economy and urbanization has experienced rapid growth, which significantly changed the transportation modes in China[1]. A total of 203,049 road traffic accidents occurred in 2017 in China, which caused 63,772 fatalities, 209,654 injuries, and direct economic loss valued at 1.21 billion yuan[2]. Traffic accidents can not only threaten road users’ lives, but also lead to road traffic congestion and other traffic issues. Therefore, to reduce the occurrence of traffic accidents and the impact of traffic accidents on road traffic, it is vital to analyze the important factors that affect the occurrence of traffic accidents and to put forward the corresponding accident analyzing model. This can provide not only a scientific basis for traffic management to optimize the road environment, but also useful information for the development of scientific traffic regulations. As a consequence, many methodologies are used to investigate the relationship between the risk factors and traffic accident frequency occurring at certain road entities (e.g. road segments or intersections) over some specified periods (e.g. weeks, months or years), such as Poisson regression[3], Poisson model’s variations[4–8]. Besides, some other methods, such as negative binomial regression model[9], mixed logit model[10], artificial intelligence models[11–14], osculating value method[15], computer vision method[16], have also been used to analyze traffic safety and traffic management.

Conventional traffic accident models assume that traffic accidents are independent, so traffic accidents occurring at different locations are not related. However, the spatial dependence of traffic accidents does not meet this assumption. This dependency mainly comes from two aspects. Firstly, in general, the road traffic flow of central urban areas is larger than that of suburbs, so the probability that traffic accidents occurring at a central business district is larger than that of surrounding suburban areas. With the increased distance from the city center, the number of accidents also reduce sharply. Secondly, the road environment has a significant effect on the traffic accident, and those roads that are close to each other have similar road conditions. For instance, roads in close proximity have similar traffic flow characteristics, so the accident is often clustered together. Therefore, the traffic accident has spatial correlation, and the traditional accident model is no longer applicable. The spatial model, however, can address the spatial dependence factor, and is an effective method to deal with spatial interaction (spatial autocorrelation) and spatial structure (spatial inhomogeneity). Appropriate consideration of spatial dependency is helpful in adjusting the impact of accidental factors in the statistical model and making the model more accurate. Many previous researches found that spatial methods are capable of accounting for the spatial correlation in crash data from the macro perspective and the spatial methods outperform the non-spatial models[17,18].

Spatial model originated from Besag[19] and improved by Quddus[20], Lesage[21] and Anselin[22]. Now, spatial model is one of the basic methods in theoretical geography. Considering the existence of many spatial correlation factors in the field of transportation, the spatial model method has been applied in the field of transportation in recent years:

- Considering the influence of spatial correlation on the safety of signalized intersections, Guo et al. applied the spatial model to the analysis of the security of the signalized intersections[23];
- Based on spatial correlation, Xie et al. modeled the accident frequency of the signalized intersection[24,25];
- Based on the spatial correlation analysis theory, Wang et al. analyzed the impact of traffic congestion on traffic safety[26];
- Taking the 48325 Shanghai highway traffic accident data as an example, Sun et al. divided the highway into several fragments, and then, taking its spatial correlation into account, analyzed the traffic accident mechanism under different traffic congestion levels[27];
- Xie et al. used highway incident data from New York City and its surrounding regions before and after Hurricane Sandy to develop an incident duration model, which accounts for the spatial dependence of duration observations and investigates the impacts of a hurricane on incident duration[28];

These scholars have made positive contributions to the application of spatial correlation theory in the field of traffic accidents. However, most of these studies focused on the discovery and evaluation of spatial correlation of traffic congestion and traffic accidents. Few papers analyze the factors that affect traffic accident frequency under the consideration of spatial correlation. The objective of this paper is to propose a framework of spatial panel time-fixed effects error model that can address spatial correlation/heterogeneity and temporal correlation/heterogeneity of traffic accidents and related risk factors. As an attempt to explore spatial panel modeling in traffic accidents analysis, this study has the potential to bring new viewpoints into traffic accidents analysis.

## Spatial model

### Moran’s I test

Spatial dependence of incident durations was assessed using Global Moran’s I statistic which was first proposed by Moran[29]. The Moran’s I statistic form is defined as:

$$I=\frac{n\sum_{i=1}^{n}\sum_{j=1,j\neq i}^{n}w_{ij}\left(y_{i}-\bar{y}\right)\left(y_{j}-\bar{y}\right)}{(\sum_{i=1}^{n}\sum_{j=1,j\neq i}^{n}w_{ij})\sum_{i=1}^{n}{\left(y_{i}-\bar{y}\right)}^{2}}$$ (1)

where *n* is the total number of traffic analysis zones (TAZs), *w*_*ij*_ is the spatial weights between *i*th traffic analysis zone (TAZ) and *j*th traffic analysis zone (if *i* and *j* are adjacent *w*_*ij*_ = 1, otherwise, *w*_*ij*_ = 0), *y*_*i*_ is the crash frequency of *i*th (TAZ), and *ȳ* is the expected crash number, $\bar{y}=(\sum_{i=1}^{n}y_{i})/n$.

The *z-value* of Moran’s I is given by Eq (2):

$$z-value=\frac{I-E\left[I\right]}{SD\left[I\right]}$$ (2)

where *E*[*I*] and *SD*[*I*] are the expectation and standard deviation of *I*. A negative *z-value* implies that the observations tend to be more dissimilar than one might expect, while a positive *z-value* indicates that the distribution of the observations is spatially clustered.

Anselin et al. suggested using *pseudo p-value* obtained from permutation test to assess the significance of Moran’s I[30]. If *pseudo p-value* is less than 0.05, I is statistically significant at the confidence level of 95% and it suggests spatial dependence of the observations. On the other hand, if the *pseudo p-value* is greater than or equal to 0.05, it indicates that observations are likely to be randomly and independently distributed in space. The *pseudo p-value* can be computed by Eq (3):

$$pseudo\;p-value=\frac{M+1}{S+1}$$ (3)

where *M* is the number of instances with Moran’s I equal to or greater than that of the observed data and *S* is the total number of permutations.

### Description of spatial dependency

**Fig 1:**
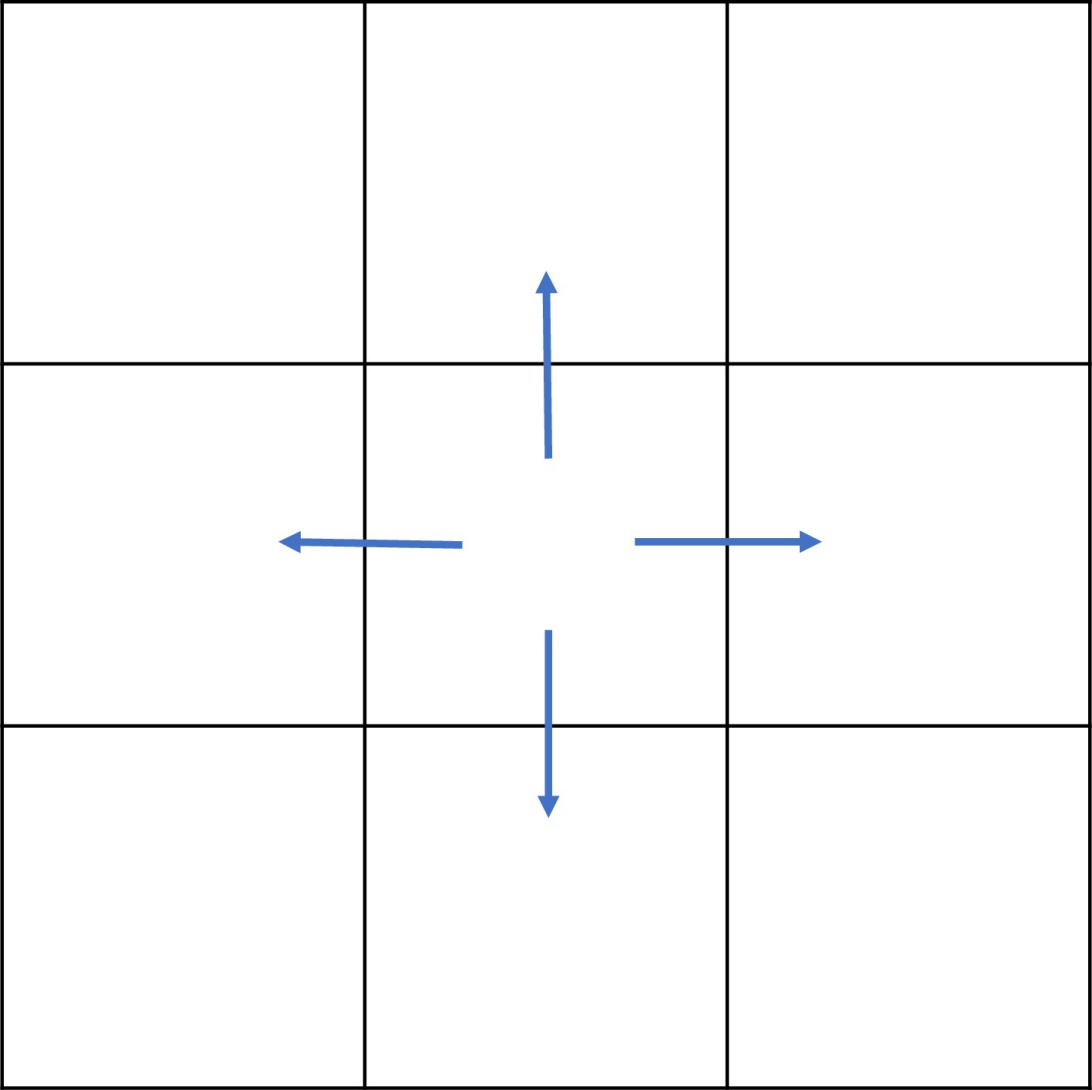
Schematic diagram of rook contiguity.

GeoDa was used to test whether the incident durations were spatially correlated. Spatial dependency is expressed by spatial weight matrix, which can be divided into two categories: "adjacency" and "distance". "Adjacency" spatial dependency that corresponds to the spatial weight matrix element value is defined as: adjacent 1; otherwise, 0. The "distance" spatial dependency is based on the distance between different regions to determine the size of its spatial weight matrix element values. This study uses the "adjacency" spatial dependency, which mainly includes "rook" contiguity and "queen" contiguity. "Rook" contiguity is defined as: if there are common edges between the two TAZs, they are defined as "adjacency". Otherwise they are defined as "not adjacent" (Fig 1).

**Fig 2:**
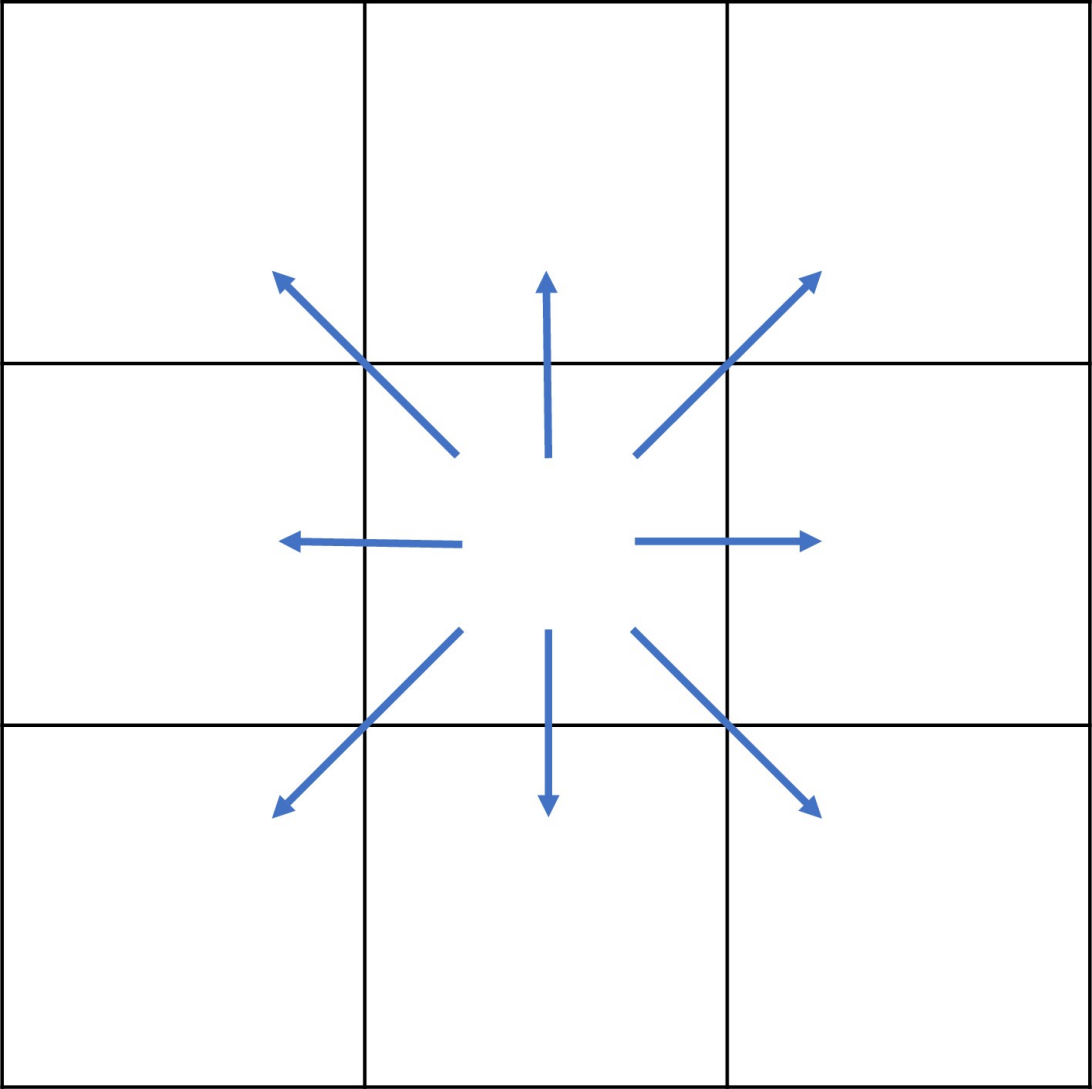
Schematic diagram of queen contiguity.

“Queen" contiguity is defined as: if there are common edges or common vertices between the two TAZs, they are defined as "adjacency". Otherwise they are defined as "not adjacent" (Fig 2).

## Modeling methodology

The standard approach in most previous researches is nonspatial linear regression model. The nonspatial linear regression model is as follows:

$$\begin{array}{l} y=\alpha +X\beta +\varepsilon ,\\ \varepsilon ∼N\left(0,\sigma^{2}I\right) \end{array}$$ (4)

where **y** is an *N × 1* vector consisting of one observation on the dependent variable for every traffic analysis zone (TAZ) (*i =* 1,*…*,*N*); α denotes the constant term; ***X*** is the matrix of explanatory variables, and there are *K* explanatory variables; ***β*** is vector of parameters; *ε* = (*ε*_*1*_,…,*ε*_*N*_)^T^ is a vector of general residual term. The linear regression model is often labeled ordinary least squares (OLS) model. Even though the OLS model in most researches on spatial methods is rejected in favor of a more general model, its results often serve as a benchmark.

### Fixed effect model and random effect model

According to whether there is a correlation between the individual effect and the independent variables, the spatial panel data model includes the fixed effect model (FEM) and the random effect model (REM).

The fixed effect model’s framework is as follows:

$$\begin{array}{l} y_{it}=\mu_{i}+X_{it}\beta +\varepsilon_{it}\\ \varepsilon_{it}∼N\left(0,\sigma_{\varepsilon }^{2}\right) \end{array}$$ (5)

where ***X***_*it*_ is the design matrix of explanatory variables, and *μ*_*i*_ is the unit-specific effect. Eq (5) can also be expressed with a dummy variable as:

$$\begin{array}{l} y_{it}=\sum_{j=1}^{N}\mu_{i}d_{ij}+X_{it}\beta +\varepsilon_{it}\\ \varepsilon_{it}∼N\left(0,\sigma_{\varepsilon }^{2}\right) \end{array}$$ (6)

where, if *i = j*, *d*_*ij*_ = 1, otherwise, *d*_*ij*_ = 0.

Random effect model’s framework is as follows:

$$\begin{array}{l} y_{it}=a+X_{it}\beta +\mu_{i}+\varepsilon_{it}\\ \varepsilon_{it}∼N\left(0,\sigma_{\varepsilon }^{2}\right) \end{array}$$ (7)

where *a* is intercept term, residual term contains *μ*_*i*_, the unit-specific effect, and *ε*_*it*_, the general residual term, and E(*μ*_*i*_|*x*_*it*_) = 0.

### Spatial regression model

The spatial correlation is set as the spatial autocorrelation form of the variable in the model. That is, the spatial correlation is introduced into the common panel data model through the spatial lag factor of the variable. At present, the spatial lag factors commonly used in the study of spatial econometrics include spatial autocorrelation of dependent variables and spatial autocorrelation of model errors. The corresponding models are spatial lag model (SLM) and spatial error model (SEM), respectively. The spatial lag model is applicable to the case where the dependent variable is directly affected by the dependent variable value of the adjacent region.

If the dependent variable and the surrounding value of the region is affected by some of the unobserved spatial aggregation features rather than directly affected by the dependent variable value of the adjacent region, the spatial error model is more suitable.

### Spatial error model

A spatial error model assumes that spatial autoregressive process occurs only in the error term. Spatial dependence is captured via spatial error correlation. The spatial error model in matrix form can be specified as:

$$\begin{array}{l} y=X\beta +\lambda Wu+\varepsilon \\ \varepsilon ∼N\left(0,\sigma^{2}I\right) \end{array}$$ (8)

where ***y*** is the vector of logarithm of incident durations, ***X*** is the vector of explanatory variables such as incident type, incident time and incident location, ***β*** is the vector of regression coefficients to be estimated, and I represents the identity matrix. The overall error is represented by two components, namely ***ε***, a spatially uncorrelated error term which is assumed to be independent and identically distributed with mean zero and constant variance, and ***u***, a spatially dependent error term. The spatial autoregressive parameter ***λ*** indicates the extent to which ***u*** of observations are correlated. The log-likelihood function for the spatial error model is:

$$\begin{array}{c} \mathrm{LL}\left(\beta ,\sigma ,\rho \right)=\ln\left|I-\lambda W\right|-\left(N/2\right)\ln\left(2\pi \right)-\left(N/2\right)\ln\left(\sigma^{2}\right)\\ -\left(1/2\sigma^{2}\right){\left(y-\lambda Wy-X\beta +\lambda WX\beta \right)}^{'}\left(y-\lambda Wy-X\beta +\lambda WX\beta \right) \end{array}$$ (9)

The maximum likelihood (ML) estimation first outlined by Ord was used to calibrate the spatial error model and the spatial lag model[31]. The ML estimators of the coefficients ${\hat{\beta }}_{ML}$ and their variance ${\hat{\sigma }}_{ML}^{2}$ in the spatial error model are given as follows:

$${\hat{\beta }}_{ML}={\left[{\left(X-\lambda WX\right)}^{'}\left(X-\lambda WX\right)\right]}^{-1}{\left(X-\lambda WX\right)}^{'}\left(y-\lambda Wy\right)$$ (10)

and

$$\begin{array}{c} \begin{array}{c} {\hat{\sigma }}_{ML}^{2}={\left(e-\lambda We\right)}^{'}\left(e-\lambda We\right)/N\\ e=y-X{\hat{\beta }}_{ML} \end{array} \end{array}$$ (11)

### Spatial lag model

A spatial lag model assumes spatial autoregressive process occurs only in the dependent variable. Spatial dependence is captured through both spatial error correlation effects and spatial spillover effects. The spatial lag model in matrix form can be specified as:

$$\begin{array}{l} y=X\beta +\rho Wy+\varepsilon ,\\ \varepsilon ∼N\left(0,\sigma^{2}I\right) \end{array}$$ (12)

where *ρ****Wy*** is a spatially lagged dependent variable, *ρ* is a spatial autoregressive parameter, and the rest of the notation is as before. The assumption of error term ***ε*** is the same as the one in Eq (8). It should be noted that if *ρ* = 0, there is no spatial dependence and the model reduces to the standard lognormal model. The log-likelihood function for the spatial error model is:

$$\begin{array}{c} \mathrm{LL}\left(\beta ,\sigma ,\rho \right)=\ln\left|I-\rho W\right|-\left(N/2\right)\ln\left(2\pi \right)-\left(N/2\right)\ln\left(\sigma^{2}\right)\\ -\left(1/2\sigma^{2}\right){\left(y-\rho Wy-X\beta \right)}^{'}\left(y-\rho Wy-X\beta \right) \end{array}$$ (13)

The maximum likelihood estimators of the coefficients ${\hat{\beta }}_{ML}$ and their variance ${\hat{\sigma }}_{ML}^{2}$ in the spatial lag model are given as follows:

$$\begin{array}{c} \begin{array}{c} {\hat{\beta }}_{ML}={\hat{\beta }}_{0}-\rho {\hat{\beta }}_{L}\\ \begin{array}{c} {\hat{\beta }}_{0}={\left(X^{\prime }X\right)}^{-1}X^{\prime }y\\ {\hat{\beta }}_{L}={\left(X^{\prime }X\right)}^{-1}X^{\prime }Wy \end{array} \end{array} \end{array}$$ (14)

and

$$\begin{array}{c} \begin{array}{c} {\hat{\sigma }}_{ML}^{2}={\left(e_{0}-\rho e_{L}\right)}^{'}\left(e_{0}-\rho e_{L}\right)/N\\ \begin{array}{c} e_{0}=y-X{\hat{\beta }}_{0}\\ e_{L}=y-X{\hat{\beta }}_{L} \end{array} \end{array} \end{array}$$ (15)

### Time-fixed effect error model

There are two kinds of spatial panel data models based on time fixed effect: the time-fixed effect lag model (T-FELM) and the time-fixed effect error model (T-FEEM).

Time-fixed effect lag model’s frame is as follows:

$$\begin{array}{l} y_{it}=X_{it}\beta +\rho Wy_{it}+\mu_{i}+\varepsilon_{it}\\ \varepsilon_{it}∼N\left(0,\sigma^{2}I\right) \end{array}$$ (16)

Time-fixed effect error model’s frame is as follows:

$$\begin{array}{c} \begin{array}{c} y_{it}=X_{it}\beta +\mu_{i}+u_{it}\\ \begin{array}{c} u_{it}=\lambda Wu_{it}+\varepsilon_{it}\\ \varepsilon_{it}∼N\left(0,\sigma^{2}I\right) \end{array} \end{array} \end{array}$$ (17)

where *i* is the index of cross section dimension, *i* = 1,…, *N*, *N* is the cross-section size, *t* is the index of time dimension, and the rest of the notation is as before.

The parameters of the model in (17) can be estimated in three steps. First, the fixed effects *μ*_*i*_ are eliminated from the regression equation by demeaning the *y* and *x* variables. This transformation takes the form

$$\begin{array}{c} \begin{array}{c} y_{it}^{*}=y_{it}-\frac{1}{T}\sum_{t=1}^{T}y_{it}\\ x_{it}^{*}=x_{it}-\frac{1}{T}\sum_{t=1}^{T}x_{it} \end{array} \end{array}$$ (18)

Second, the transformed regression equation *y*_it_^*^ *=* ***x***_it_^*^β *+ ε*_it_^*^ is estimated by OLS: ***β*** = (X*^T^X*)^-1^X*^T^Y* and *σ*^2^ = (Y^*^- X^*^β)^T^(Y^*^- X^*^β)/(*NT- N- K*), where the asterisk denotes the demeaning procedure. This estimator is known as the least squares dummy variables (LSDV) estimator.

Instead of estimating the demeaned equation by OLS, it can also be estimated by ML. Since the log-likelihood function of the demeaned equation is

$$\begin{array}{c} LL=-\frac{NT}{2}\log\left(2\pi \sigma^{2}\right)+T\log\left|I_{N}-\lambda W\right|\\ -\frac{1}{2\sigma^{2}}\sum_{i=1}^{N}\sum_{t=1}^{T}{\left\{y_{it}^{*}-\lambda {\left[\sum_{j=1}^{N}w_{ij}y_{ij}\right]}^{*}-\left(x_{it}^{*}-\lambda {\left[\sum_{j=1}^{N}w_{ij}y_{ij}\right]}^{*}\right)\beta \right\}}^{2} \end{array}$$ (19)

Given *λ*, the ML estimators of ***β*** and *σ*^2^ can be solved from their first-order maximizing conditions, to get

$$\begin{array}{c} \beta ={\left({\left[X^{*}-\lambda \left(I_{T}⊗W\right)X^{*}\right]}^{T}\left[X^{*}-\lambda \left(I_{T}⊗W\right)X^{*}\right]\right)}^{-1}\\ \times \left({\left[X^{*}-\lambda \left(I_{T}⊗W\right)X^{*}\right]}^{T}\left[Y^{*}-\lambda \left(I_{T}⊗W\right)Y^{*}\right]\right) \end{array}$$ (20)

$$\begin{array}{c} \sigma^{2}=\frac{e{\left(\lambda \right)}^{T}e\left(\lambda \right)}{NT} \end{array}$$ (21)

Where ***e***(*λ*) = Y*—*λ*(***I***_*T*_ ⊗ ***W***) Y*—[X*—*λ*(*I*_*T*_ ⊗ ***W***) X*]***β***.

Finally, the fixed effects can be estimated by

$$\begin{array}{c} \mu_{i}=\frac{1}{T}\sum_{t=1}^{T}\left(y_{it}-x_{it}\beta \right) \end{array}$$ (22)

More detailed descriptions and assessments of these models can be found in the papers of Elhorst[32,33].

### Model assessment

The coefficient of determination *R*^2^ is usually used to measure the goodness-of-fit of models. However, it is inappropriate to use *R*^2^ to assess spatial models because the residuals of spatial models are not independent of one another. This issue can be appropriately addressed by using criteria based on likelihood estimation, such as maximum likelihood and Akaike Information Criterion (AIC) which was proposed by Akaike. In addition, AIC can serve as a comprehensive measure of model fitting and model complexity by introducing parameter number as a penalty term. As an alternative to AIC, Bayesian Information Criterion (BIC) combines the parameter number and the sample size into the penalty term. The AIC and BIC can be expressed as:

$$\begin{array}{c} \mathrm{AIC}=-2{\mathrm{LL}}_{\max}+2k \end{array}$$ (23)

$$\mathrm{BIC}=-2{\mathrm{LL}}_{\max}+k\ln\left(N\right)$$ (24)

where LL_max_ is the maximum of log-likelihood that can be obtained using Eqs (9) and (13), *k* is the parameter number and *N* is the sample size. The LL_max_, AIC and BIC were used to compare the models with difference specifications. If the AIC and BIC differences between two models are greater than four, the two models can be regarded as considerably different; if the differences are greater than 10, it provides strong evidence that the model with a lower AIC and BIC should be favored.

## Accident data

### Data collection

Data sets were collected from Guiyang City, China during the year 2015. Guiyang City is a large city located in the southwestern part of China. It is the provincial capital of the Guizhou province. The accident data set only includes traffic accidents involving motor vehicles or motorcycles. There was a total of 56,652 traffic accidents that occurred in the city and the city’s surrounding area in 2015. After the removal of abnormal data, there were 308 days with a total of 53,592 accident records left. There were 174 accidents that occurred per day on average during 2015. The data set includes accident features (date, time etc.), accident location, limited personal attributes of the drivers (age, gender etc.) and accident type.

**Fig 3:**
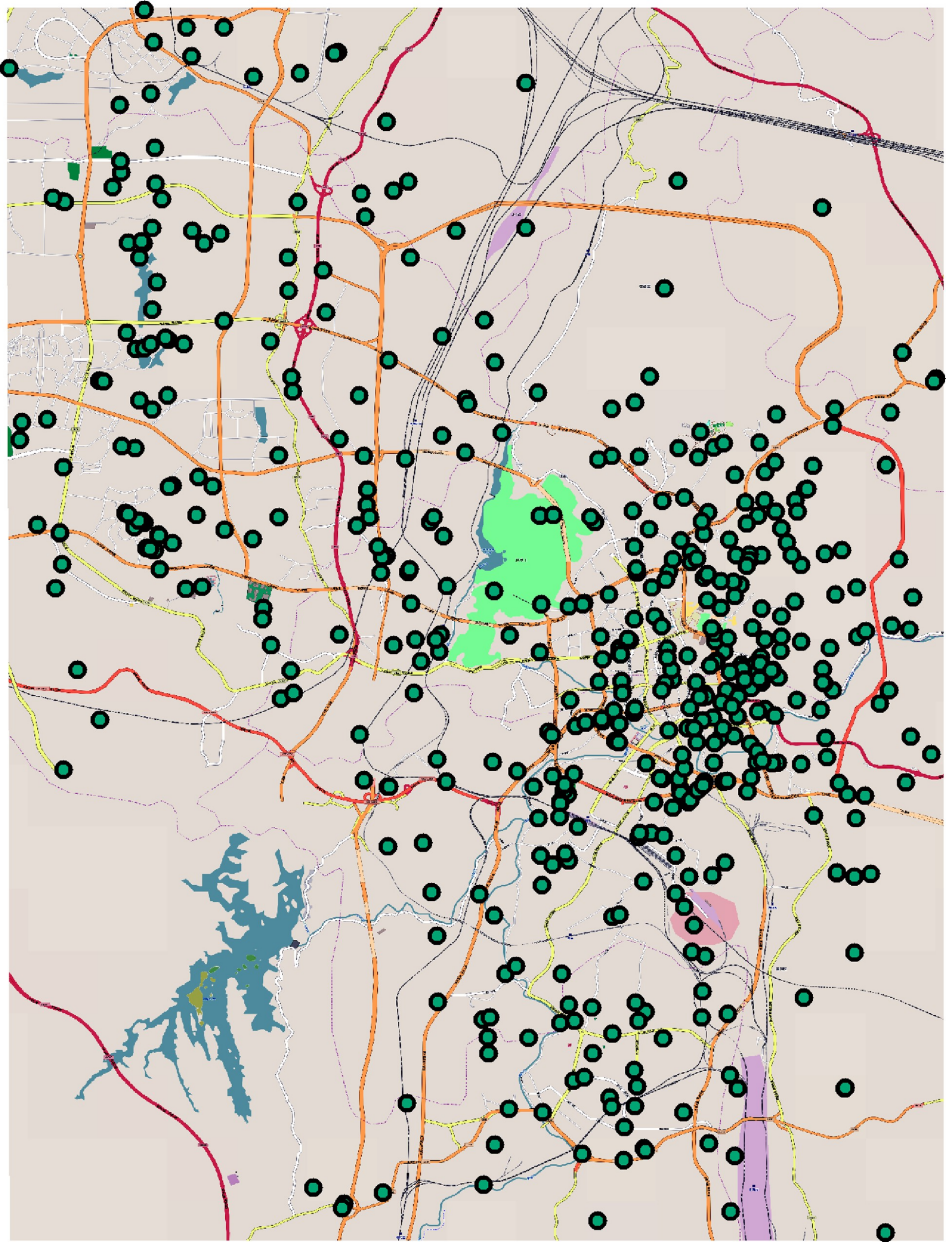
Schematic diagram of location of traffic accidents.

According to the description of the location in the accident record, we found the latitude and longitude of the site from the Internet (http://apistore.baidu.com/). We complied with the terms of service for the websites from which they collected data. As the number of accidents occurred in the suburbs of the city is relatively less, we intercepted an area located in the inner part of the ring freeway (about 22.08km in the east-west direction and about 15.48km in the north-south direction). To improve the accuracy of the data set used in this study, accident records with vague accident locations were removed, as those data points could not be used to determine the precise location. The schematic diagram of location of traffic accidents is shown below (Fig 3).

**Fig 4:**
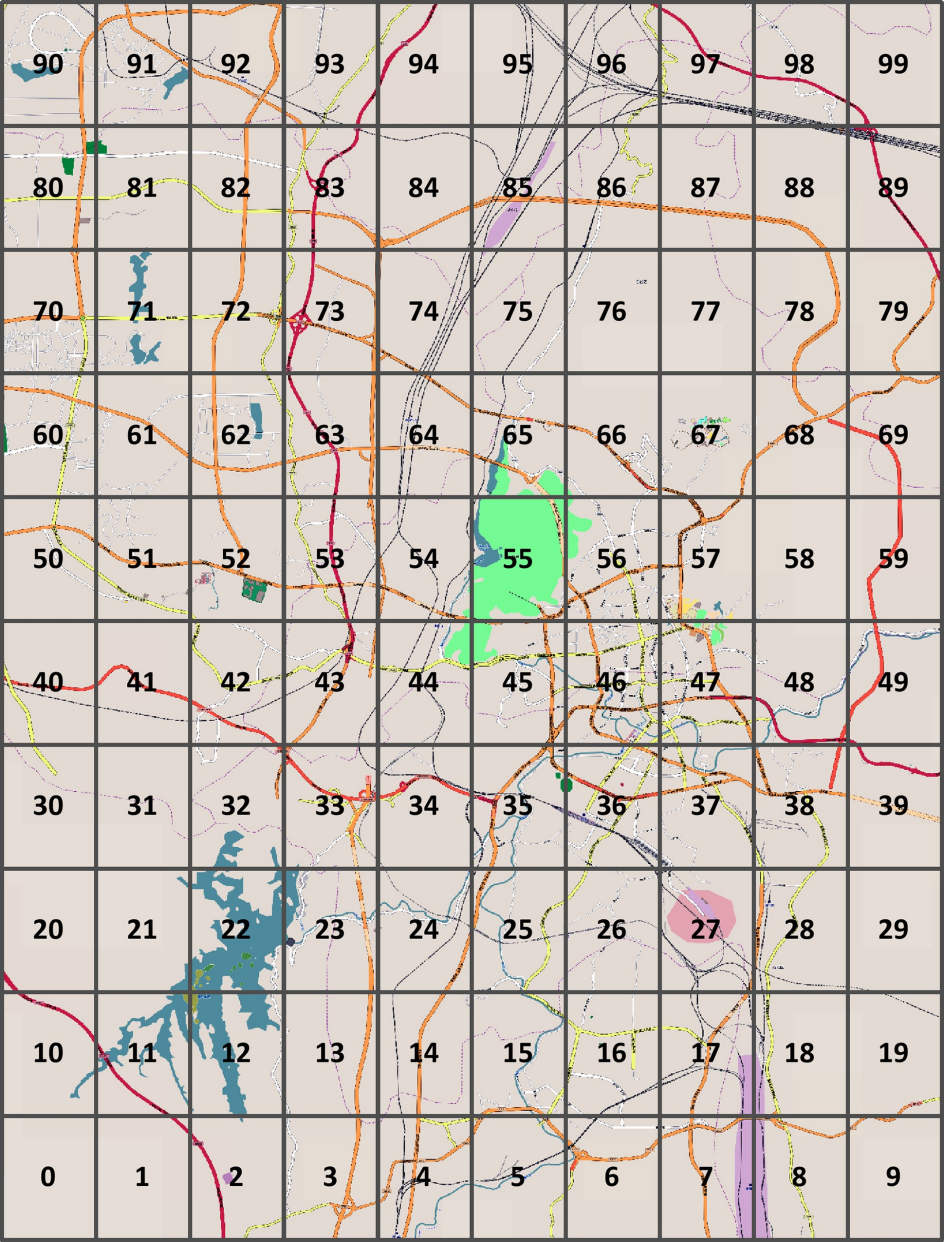
Schematic diagram of cells coding.

ArcGIS was applied to merge the traffic accident data and city boundary. The final data was verified and checked for errors and reasonableness. To visualize the spatial distribution of the number of accidents, and to build the traffic accident spatial model, we split the study area into 100 equally sized rectangular traffic analysis zones (TAZs) (2.208 km × 1.548 km) in the GIS map and computed the number of accidents located in each TAZ. 100 TAZs are numbered 0–99, as shown below (Fig 4).

### Descriptive analyses of incident data

Many previous researches found that inner ring or outer ring (urban or suburban)[34–36], the presence of highway[37] and presence of school (or number of school)[38,39] have associations with the crash frequency. According to people’s intuition, areas have hospital, passenger station or flyover tend to have higher exposure of pedestrians and higher traffic volume, and traffic accidents are more likely to occur in these areas. In the light of the above discussion, six TAZ attributes were chosen as factors affecting traffic accidents. They are: TAZ location (whether it is located in the second ring freeway), whether there is a freeway (Inn_sec_ring) through (Freeway), number of hospitals (N_hospital), number of schools (N_school) and whether there is a passenger station (Pass_station) and the presence of flyover (Flyover). The description of each variable and its descriptive statistics are shown in Table 1.

**Table 1:** Description and descriptive statistics of incident data.

Variables	Description	Mean	S.D.
N_accident	Total number of crashes per TAZ	75.56	141.33
**Attributes of TAZ**			
Inn_sec_ring	1 if TAZ is located in the inner second ring freeway area, 0 otherwise	0.30	0.46
Freeway	1 if at least one freeway passes through this TAZ, 0 otherwise	0.46	0.50
N_hospital	Number of tertiary hospital	0.15	0.50
N_school	Number of primary school and secondary school	1.11	1.94
Pass_station	1 if at least one passenger station in this TAZ, 0 otherwise	0.06	0.24
Flyover	1 if at least one flyover in this TAZ, 0 otherwise	0.27	0.45

**Fig 5:**
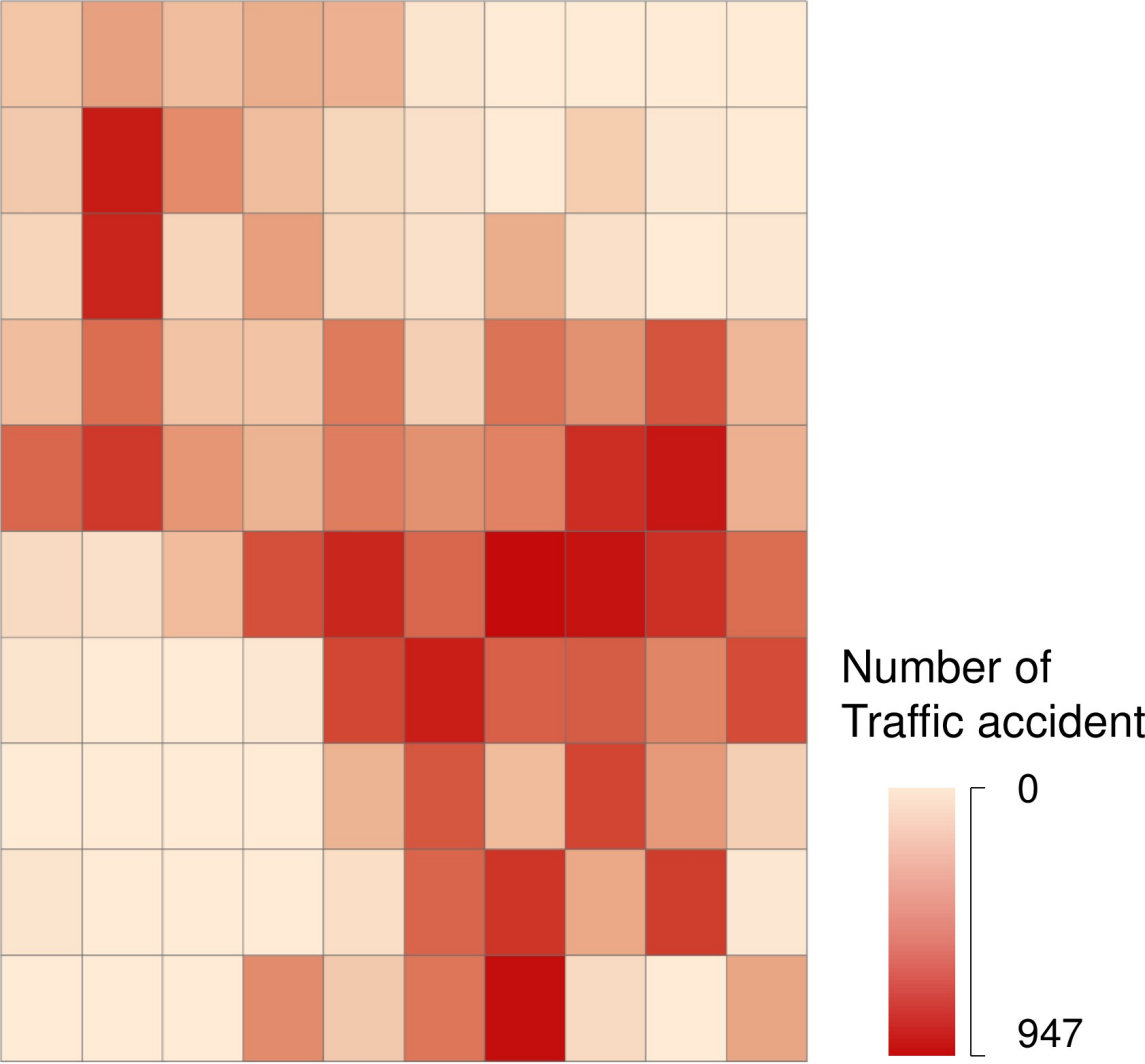
Spatial clustering of traffic accidents.

To get a more imaginable spatial distribution of traffic accident frequency, number of accidents of each zone is computed and five-point bitmap of distribution of traffic accident number is drawn (Fig 5). In Fig 5, the number of accidents in each TAZ is expressed by the color of different depths. The darker the color is, the more traffic accidents occur in the TAZ. We can see the spatial clustering of TAZs of the same color. For example, lighter-colored TAZs are concentrated in the lower left and upper right corners of the area, and the darker TAZs are centered on the middle right of the area. This indicates that the number of traffic accidents in adjacent areas of the study area is close, that is, the number of accidents has spatial correlation.

## Results

### Moran’s I test

Spatial weight matrices of 100 TAZs are established by GeoDa, which have adjacency rules of 1st order "queen" contiguity and 1st order "rook" contiguity, respectively. The results of Moran’s index I are shown in Table 2. All p values obtained by permutation using 1st order "queen" contiguity and 1st order "rook" contiguity are below 0.05, which indicates that traffic accident frequency does have spatial dependence. If the spatial dependence of the adjacent area occurring traffic accident is ignored, it will lead to an estimated deviation.

**Table 2:** Results of Moran’s I test.

	Permutations	I	E[I]	SD[I]	z-value	pseudo p-value
**queen**	499	0.2392	-0.0101	0.0522	4.8652	0.002
	999	0.2392	-0.0101	0.0535	4.7124	0.003
**rook**	499	0.2498	-0.0101	0.0727	3.5816	0.004
	999	0.2498	-0.0101	0.076	3.4223	0.003

### Models for accident

This part compares the general model without considering spatial dependence, and SEM and SLM considering spatial dependence. The parameter estimation of the general model is based on the least squares method, while parameter estimation of SLM and SEM are the maximum likelihood estimation method. Using ArcGIS to calculate the ratio of different incidents in each TAZ, combined with the number of hospitals in the TAZ, the number of schools and other attributes, get it and graphics files together with GeoDa. Taking Inn_sec_ring, N_hospital, N_school, Pass_station, Freeway, and Flyover as independent variables, “common” least square regression model, SLM, and SEM are established (Table 3).

**Table 3:** Result of traffic accidents models.

Models	OLS			SLM			SEM			T-FEEM		
Variable	Coefficient	Std. Error	Probability	Coefficient	Std. Error	Probability	Coefficient	Std. Error	Probability	Estimate	Std. Error	Probability
Constant	40.026	25.206	0.116	32.543	25.512	0.202	36.413	22.639	0.108			
Inn_sec_ring	47.133	38.573	0.225	31.297	39.152	0.424	48.850	35.180	0.165	4.319	1.791	0.016
Freeway	9.008	37.597	0.811	8.589	35.996	0.811	8.159	35.461	0.818	0.811	1.682	0.630
N_hospital	264.154	38.595	0.000	259.218	37.076	0.000	267.563	36.870	0.000	26.055	1.644	0.000
N_school	14.551	10.168	0.156	11.671	9.852	0.236	16.212	9.630	0.092	1.154	0.444	0.009
Pass_station	-244.028	74.779	0.002	-254.073	72.034	0.000	-242.585	72.357	0.001	-21.305	3.249	0.000
Flyover	24.507	42.873	0.569	25.644	41.107	0.533	28.906	40.495	0.475	2.407	1.870	0.198
W_ N_accident				0.143	0.129	0.267						
λ							-0.115	0.171	0.503			
ρ										0.280	0.050	0.000
**Model assessment**												
R^2^	0.471			0.479			0.474			0.838		
AIC	1312.080			1312.950			1311.790			225.463		
SC	1330.320			1333.800			1330.020			240.372		

In addition, traffic accident frequency in different TAZs is calculated by month, from March to December for a total of 10 months. Considering the correlation of time and space, the data is analyzed by the spatial panel data’s analysis method. Spatial panel data models include T-FEEM, T-FELM, individual random effect error model(IREEM) and so on. In order to determine the optimal model, this part first uses the Baltagi test to test whether there are spatial lag correlation and random effect in panel data. The result of spatial lag correlation is: SLM1 = 0.024879, p-value = 0.9802 (rook associated), p-value is more than 0.05, which shows that null hypothesis is agreed, or there is no spatial lag correlation in panel data. The result of random effect is: SLM2 = 0.01199, p-value = 0.9904 (rook associated), p-value is more than 0.05, which shows that null hypothesis is agreed, or there is no random effect in panel data.

According to the test results, this part finally selects T-FEEM based on rook correlation as the model to be established for the analysis of the spatial panel. Taking the amount of traffic accidents in different TAZs in different time periods as dependent variables, and taking Inn_sec_ring, N_hospital, N_school, Pass_station, Freeway, and Flyover as independent variables, the model result is obtained by maximum likelihood estimation. The established “common” least square regression model, SLM, SEM, and T-FEEM are compared, which have results shown in Table 3.

From Table 3, we can clearly see that the ACI and BIC obtained by the T-FEEM are the lowest, which helps conclude that the T-FEEM is better than other models. What’s more, it also can be seen from Table 3 that SEM is superior to the SLM. That means the spatial dependence of accident frequency is derived mainly from the attributes of the adjacent TAZs and some other unobserved factors. Hence, this paper applies the T-FEEM to evaluate the influences of different independent variables on the traffic accidents. And in Table 3, if the coefficient of independent variables is positive, it means that the increase of the independent variable will lead to the increase of the area’s traffic accidents. On the contrary, if the coefficient of the independent variable is negative, the increase of the independent variable will lead to the decrease of the area’s traffic accidents.

In addition, in Table 3, four variables (i.e., Inn_sec_ring, the number of hospitals, the number of schools and the passenger stations) of the T-FEEM have a significant effect on the traffic accident (i.e., p = 0.05), and the significance test values of freeway passing and flyover variables are higher than 0.05, which have no significant effect on the occurrences of area traffic accident.

The p-value of Inn_sec_ring is less than 0.05, indicating that this variable has a significant effect on traffic accident frequency. If the coefficient of this variable is positive, it indicates that the average number of accidents of the TAZs within the second ring freeway is higher than that of the district outside the second ring freeway. It is consistent with previous study [34–36]. Specifically, the accidents in the inner area are 4.3 times greater than that of the outside area of the second ring freeway. The reason is that the traffic flow, especially the non-motor vehicle and pedestrian traffic flow in the Inn_sec_ring, are more than those of the area outside of second ring freeway, which unavoidably have a higher probability of conflict among vehicles, motorcycles, cycles and pedestrians and lead to more traffic accidents.

As for the number of hospitals, the higher the number of hospitals, the more traffic accidents in the TAZs. Similar to other situations, when there is an additional tertiary hospital, 26 more accidents will occur in that TAZ. This is mainly due to massive traffic flows near the hospital, poor traffic order and more traffic flow conflicts, which lead to a greater chance of traffic accidents.

The same as the influence of the number of hospitals, higher number of schools lead to more traffic accidents in the TAZs. This finding conforming with many previous research results[38,39]. This is mainly due to the rapid increase of traffic flow near the school during the school hours, leading to increases of traffic flow conflict. However, it is different from the impact of the number of hospitals, where there is an additional school, only one more accident will occur in that TAZ, which is far less than the 26 accidents caused by the increase of hospitals. There are mainly three reasons leading to this result:

1. Guiyang City, where there are many high-quality hospitals, is the provincial capital of the Guizhou province. More hospitals are generally associated with more attractions, stores, which in general indicate more traffic flow and pedestrians. This eventually could increase the probability of crashes.
2. Primary and secondary schools only have a large demand for traffic during ongoing school hours and after school hours, during which parking demands are lower and parking times are shorter. However, the large hospitals usually have consistent traffic demand throughout the day, which always leads to a higher parking demand and a need for longer parking time. Moreover, due to insufficient parking facilities, road traffic queuing and congestion usually happens near the hospital, causing further traffic accidents.
3. Most of the pedestrians and non-motor vehicle drivers near the school are the attending students and their parents, who generally comply with traffic laws and regulations, leading to a much better traffic order. For the hospital, due to the variety of reasons for visiting, the patients usually come from different parts of the province with varying backgrounds in driving experience. As a result, non-compliance of traffic rules often occurs, leading to heavy and less orderly traffic.
4. Patients who go to the hospital are often in a hurry, and maybe emotional and anxious. Due to this factor, the motor vehicle drivers are more likely to not comply with traffic rules, which increases the possibility of traffic accidents.

Whether there exists a passenger station has a significant effect on the amount of traffic accidents. A region that has large passenger stations usually has fewer traffic accidents, which contradicts with what might be expected in an area with a higher volume of people. This may be because large passenger stations are usually located in the periphery of the city. Because of the low overall traffic volumes of those TAZs, there are fewer traffic accidents. Many previous studies had found that the urban variable is expected to have a positive association with the crash frequency[34,35]. In other way, urban area has more traffic accidents than suburban area. These results can account for the passenger station variable in some extent.

## Conclusions

This study advocates a spatial panel time-fixed effects error model for analyzing traffic accident frequencies in TAZs, which accommodates spatial correlation between adjacent TAZs and temporal correlation across observations simultaneously. First, this paper introduced the spatial model and selected 10-month traffic accident data in the area located in the inner ring freeway. Second, using ArcGIS, the accidents’ locations are located on the map, and the study area was divided into 100 equal-area rectangular cells where we extracted the traffic accident frequency and the spatial attributes of each TAZ. Third, using GeoDa to plot the number of accident distributions, the results showed that the number of accidents has spatial correlation. Fourth, the spatial weight matrix of each TAZ was further established, and the Moran's I, under different adjacency rules, was also calculated. Then, the p values obtained by all permutation tests were lower than 0.05, which further confirmed that the number of accidents had spatial dependency. At last, assuming that the number of accidents is subject to the normal distribution, this paper formulated T-FEEM for the number of accidents, which was further compared with the OLS regression model, the spatial lag model and the spatial error model. The results showed that T-FEEM is superior to the spatial lag model and the OLS regression model. Based on the above analysis, the following conclusions are further obtained:

1. In the significant level of 0.05, the Inn_sec_ring, N_hospital, N_school, and Pass_station have a significant role in traffic accident frequency.
2. Generally, the more number of hospitals and schools the TAZ has, the more traffic accidents occur in the TAZ. However, the number of hospital has much greater impact on traffic accident frequency than the number of schools. Similar to other situations, when there is an additional tertiary hospital, 26 more accidents will occur in that TAZ. Moreover, when there is an additional school, only one more accident will occur in that TAZ. This is mainly due to the fact that the impact of large tertiary hospitals is much greater than that of schools, both in terms of scope and population participation.
3. The variable of large passenger stations has a reduced effect on traffic accident frequency. The traffic accident frequency of the TAZ with large passenger stations is less than that of the TAZ without large passenger station. This may be caused by the fact that large passenger stations are always located in the periphery of the city with less total traffic flow, which contribute to less overall number of accidents.

This study contributes to the growing body of research on region traffic safety as it provides a better understanding of the contributing factors related to urban infrastructure to crash counts in different urban regions, which is also helpful to distribute traffic police and patrol vehicles more properly and to use human resource more efficiently. There still exists some shortcomings due to the complicities of the road traffic accident problem. In the future, we can try more spatial adjacency methods between the traffic accidents’ TAZs so as to determine the optimal spatial weight matrix. What’s more, the influence of the variable large passenger stations on traffic accident frequency is inconsistent with common sense. In future studies, we can further divide the statistical community in more detail to reduce the interference caused by other factors. In addition, the network density of each TAZ and the other key variables can be further considered to improve the accuracy of the model.
